# Author Correction: Potential fields and fluctuation-dissipation relations derived from human flow in urban areas modeled by a network of electric circuits

**DOI:** 10.1038/s41598-023-28522-2

**Published:** 2023-01-25

**Authors:** Yohei Shida, Jun’ichi Ozaki, Hideki Takayasu, Misako Takayasu

**Affiliations:** 1 Department of Mathematical and Computing Science, School of Computing, Tokyo Institute of Technology, 4259 Nagatsuta‑cho, Midori‑ku, Yokohama 226‑8503 Japan; 2 Institute of Innovative Research, Tokyo Institute of Technology, 4259 Nagatsuta‑cho, Midori‑ku, Yokohama 226‑8503 Japan; 3 Sony Computer Science Laboratories, 3‑14‑13 Higashi‑Gotanda, Shinagawa‑ku, Tokyo Japan

Correction to: *Scientific Reports* https://doi.org/10.1038/s41598-022-13789-8, published online 15 June 2022

The Supplementary Information file published with this Article contained an error in Table S1, where the value “1 × 10^6^” was incorrectly given as “1 × 10^−6^”. The original Supplementary Information file is provided below.

This error has now been corrected in the Supplementary Information file that accompanies the original Article.

## Supplementary Information

Supplementary Information.

