# Supplementary material for: Potential fields and fluctuation-dissipation relations derived from human flow in urban areas modeled by a network of electric circuits
Source: Sci Rep. 2022 Jun 15;12:9918. doi: 10.1038/s41598-022-13789-8 (PMC9200729; doi:10.1038/s41598-022-13789-8)
Supplement: Supplementary file 1 — Supplementary Information. [file 41598_2022_13789_MOESM1_ESM.pdf]

Potential fields and fluctuation-dissipation relations derived from human flow in urban areas modeled by a network of electric circuits

Supplementary Material

Yohei Shida[1], Jun'ichi Ozaki[2], Hideki Takayasu[2,3], and Misako Takayasu[1,2]

[1]*Department of Mathematical and Computing Science,*
School of Computing, Tokyo Institute of Technology,
4259 Nagatsuta-cho, Midori-ku, Yokohama 226-8503, Japan

[2]*Institute of Innovative Research, Tokyo Institute of Technology,*
4259 Nagatsuta-cho, Midori-ku, Yokohama 226-8503, Japan and

[3]*Sony Computer Science Laboratories,*
3-14-13 Higashi-Gotanda, Shinagawa-ku, Tokyo, Japan

(Dated: November 28, 2022)

CONTENTS

I. Parameters of Adam (A Method for Stochastic Optimization) 3

II. Spatial configuration maps of rotation around Tokyo 4

A. Spatial configuration maps of rotation around Tokyo at the initial iteration 4

B. Spatial configuration maps of rotation around Tokyo after optimization 4

III. Derivation of the discretized Poisson's equation of the electric network 5

IV. The effect of the boundary condition on the potential calculation 6

V. The results around Osaka and Nagoya metropolitan areas 8

A. The results around Osaka metropolitan area 8

B. The results around Nagoya metropolitan area 10

VI. Fluctuation-dissipation relation for 3 cities 12

References 14

I. PARAMETERS OF ADAM (A METHOD FOR STOCHASTIC OPTIMIZATION)

To minimize the sum of the rotations, L in Eq. 3 in the main text, with the restriction that each resistance is positive, we introduce a technique by adding a penalty term as follows:

$$\begin{aligned} \text{minimize} \quad L &= \textstyle\sum_{i,j,k} (\nabla \times (\boldsymbol{I}R)_{i+0.5,j+0.5,k})^2 - \gamma \sum \log R_{i,j,k} \\ \text{subject to} \quad \textstyle\sum R^0_{(i,j),(i',j')} &= \textstyle\sum R^\tau_{(i,j),(i',j')}, \end{aligned} \tag{1}$$

where $\log(\boldsymbol{I}R)$ is a logarithmic barrier function whose derivative value increases to infinity as $\boldsymbol{I}R$ reaches zero. This was done to avoid the case where R enters the negative region[1]. Here, γ is a hyperparameter that controls the influence of the barrier function. The number of resistors $R_{(i,j),(i',j')}$ and current $\boldsymbol{I}_{(i,j)\to(i',j'),k}$ in this circuit model for urban areas of Tokyo are approximately 75000 and 1500000, respectively. To solve the optimization problem with a large number of variables, we adopt Adam (abbreviation for adaptive moment estimation)[2]—a type of steepest descent method. The feature of this optimization algorithm is that the moving averages of both the past gradient ζ and the quadratic moment of the gradient η are stored, and the learning rate is automatically optimized for each variable. The τ-th update for $R_{(i,j),(i',j')}$ is given as:

$$\begin{aligned} \zeta^\tau_{(i,j),(i',j')} &= \frac{\beta_1 \zeta^{\tau-1}_{(i,j),(i',j')} + (1-\beta_1) \left.\frac{dL}{dR_{(i,j),(i',j')}}\right|_{R_{(i,j),(i',j')}=R^{\tau-1}_{(i,j),(i',j')}}}{1-\beta_1^\tau} \\ \eta^\tau_{(i,j),(i',j')} &= \frac{\beta_2 \eta^{\tau-1}_{(i,j),(i',j')} + (1-\beta_2) \left(\left.\frac{dL}{dR_{(i,j),(i',j')}}\right|_{R_{(i,j),(i',j')}=R^{\tau-1}_{(i,j),(i',j')}} \right)^2}{1-\beta_2^\tau} \\ R^\tau_{(i,j),(i',j')} &= R^{\tau-1}_{(i,j),(i',j')} - \alpha \frac{\zeta^\tau_{(i,j),(i',j')}}{\sqrt{\eta^\tau_{(i,j),(i',j')}+\epsilon}}, \end{aligned} \tag{2}$$

where $\left.\frac{dL}{dR_{(i,j),(i',j')}}\right|_{R_{(i,j),(i',j')}=R^{\tau-1}_{(i,j),(i',j')}}$ represents the derivative at $R^{\tau-1}_{(i,j),(i',j')}$. The initial values ζ^0 and η^0 of ζ and η were both 0. The values of the parameters β_1, β_2, and ϵ are given in Tab. S1, which are the standard values of this method[2]. The values of α and γ are set by trial-and-error so that the iteration converges quickly.

TAB. S1: The parameters of Adam for estimating values of conductance.

Parameters	values
β_1	0.9
β_2	0.999
ϵ	1×10^{-12}
γ	1×10^{6}
α	3×10^{-3}

II. SPATIAL CONFIGURATION MAPS OF ROTATION AROUND TOKYO

A. Spatial configuration maps of rotation around Tokyo at the initial iteration

Figs. S1a, S1b and S1c show spatial configuration maps of rotation around central Tokyo in the morning (7:30-8:00), afternoon (13:30-14:00), and evening (18:30-19:00) at the initial iteration $\tau = 0$ (i.e., the case of uniform resistance). At any time, we can find that high rotation values of opposite signs are located along the railways and highways, which are caused by large currents.

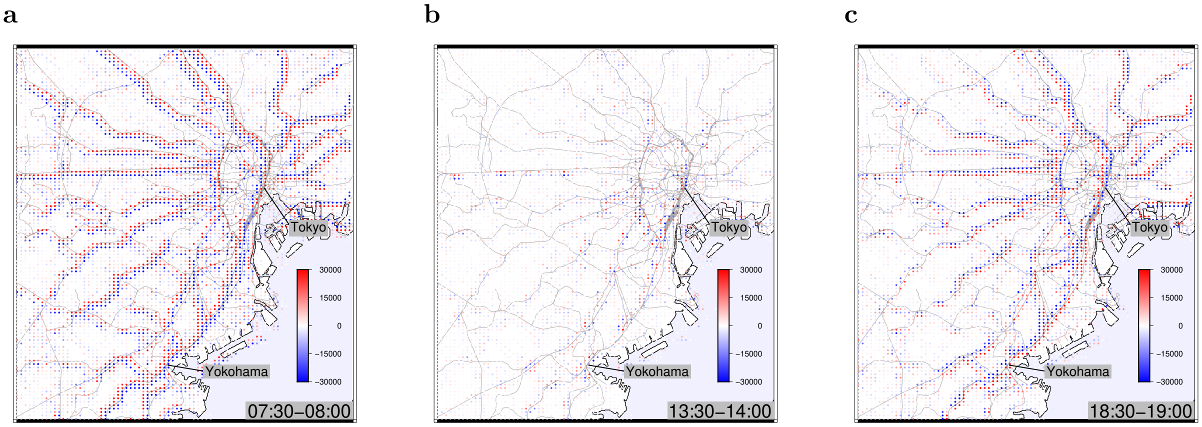

FIG. S1: Spatial configuration maps of rotation around the center of Tokyo in the morning (7:30-8:00), afternoon (13:30-14:00), and evening (18:30-19:00) at the initial iteration $\tau = 0$

B. Spatial configuration maps of rotation around Tokyo after optimization

Figs. S2a, S2b and S2c show spatial configuration maps of rotation around the center of Tokyo in the morning (7:30-8:00), afternoon (13:30-14:00), and evening (18:30-19:00) after optimization. After the optimization of the resistance values, which are independent of time, we see that the high rotation values caused by the large current is diminished and

small positive and negative values are distributed nearly uniformly for all time intervals. Note that the value on the color bar is 30 times larger than those of Fig. S1.

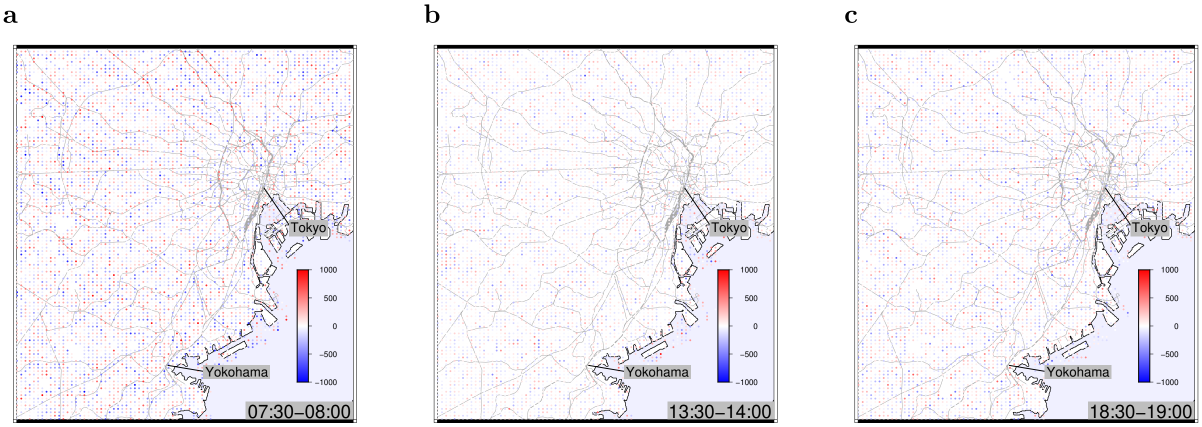

FIG. S2: Spatial configuration maps of rotation around the center of Tokyo in the morning (7:30-8:00), afternoon (13:30-14:00), and evening (18:30-19:00) after optimization

III. DERIVATION OF THE DISCRETIZED POISSON'S EQUATION OF THE ELECTRIC NETWORK

We derive the discretized Poisson's equation of the electric network according to the following procedure[3]: First, the Poisson's equation is given as:

$$\left(\nabla \cdot (\nabla \phi)\right)_{i,j,k} = (\nabla \cdot \boldsymbol{E})_{i,j,k} = Q_{i,j,k}, \tag{3}$$

where $\boldsymbol{E}$ is the vector field $\boldsymbol{I}R$, $Q_{i,j,k}$ denotes the sink or source of charges in cell (i,j) at time interval k, and $\phi_{i,j,k}$ is the electric potential at the same cell. In the following, for simplicity of notation, we omit the time variable k. We derive a discretized Poisson equation as:

$$\left(\nabla \cdot (\nabla \phi)\right)_{i,j} = Q_{i,j} = \sum_{i',j'} A_{(i,j),(i',j')}(\phi_{i',j'} - \phi_{i,j}), \tag{4}$$

where $A_{(i,j),(i',j')}$ is an element of the adjacency matrix. With respect to the coordinates (i,j), the matrix element is 1 if (i',j') is adjacent to the right (east) or top (north), and -1 if it is adjacent to the left (west) or bottom (south). Otherwise, the matrix element is 0.

The area surrounded by the red and purple lines in Fig. S3a represents the region S_1 that we work in; this coincides the government's definition of Tokyo metropolitan area[4]. The

red line shows the land boundary, which includes less populated regions such as rivers and mountain ridges. Furthermore, we fix the values of electric potential to 0 on this boundary when considering the flux across the boundary. The purple line shows the boundary between the sea and land, where we assume no flux across the boundary, while the values of electric potential are not fixed. This boundary condition is necessary because railroads and highways are often located along the coastlines, so there are high flows along the boundary; therefore, the values of potential should be different along the purple boundary. Using the National land numerical information[5], we categorize a cell as a sea cell if the area of land in the cell is less than 5%.

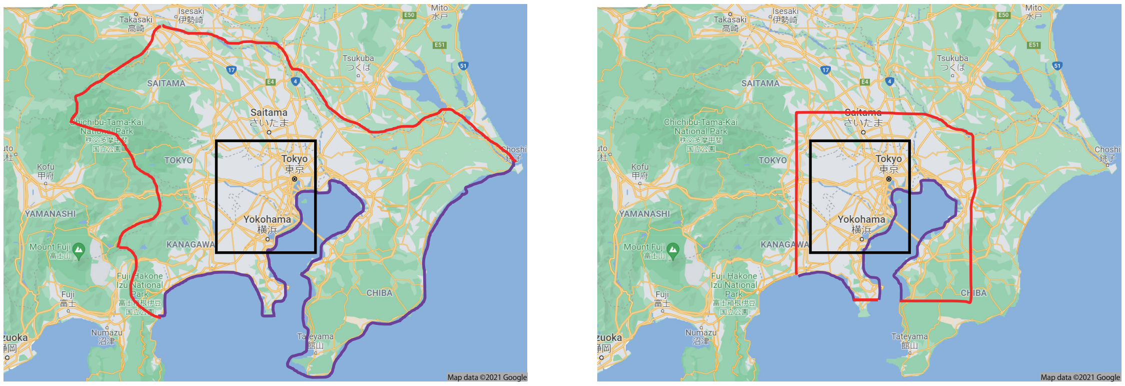

(a) (b)

FIG. S3: (a) The area we analyze here is the greater Tokyo area[4]. Tokyo and the neighboring three prefectures S_1 are shown surrounded by red and purple lines. (b) The area surrounded by the red and purple lines is defined as S_2. (a-b) The black square is S_3, which shows the figure's drawing area in the main text.

IV. THE EFFECT OF THE BOUNDARY CONDITION ON THE POTENTIAL CALCULATION

We discuss the effects of the boundary conditions on the potential calculation. To do so, we compared two boundaries, as shown in Figs. S3a and S3b. The outermost boundary S_1, which includes approximately 29000 cells, is shown in Fig. S3a represents the entire metropolitan area of Tokyo. All figures of electric potentials shown in the main text are from the black square region S_3, calculated using this boundary condition. To confirm the boundary effect, we define a smaller boundary condition S_2, as shown in Fig. S3b (which includes about 12000 cells), where red lines show the land boundary and the purple lines

show the land-sea boundary. S_3 is the center of the Tokyo metropolitan area and is also the drawing range of the figures in the main text. We calculate the electric potentials separately for S_1 and S_2 and compared the results. The difference in the potential value depending on whether the boundary is S_1 or S_2 is approximately 0.5% in the area of S_3 during the morning rush hour. We judge that the difference caused by the different boundary conditions is not effective in the center of the city, S_3.

V. THE RESULTS AROUND OSAKA AND NAGOYA METROPOLITAN AREAS

In addition to the Tokyo metropolitan area, Figs. S4-9 are results from Osaka and Nagoya metropolitan areas in the morning, afternoon, and evening.

A. The results around Osaka metropolitan area

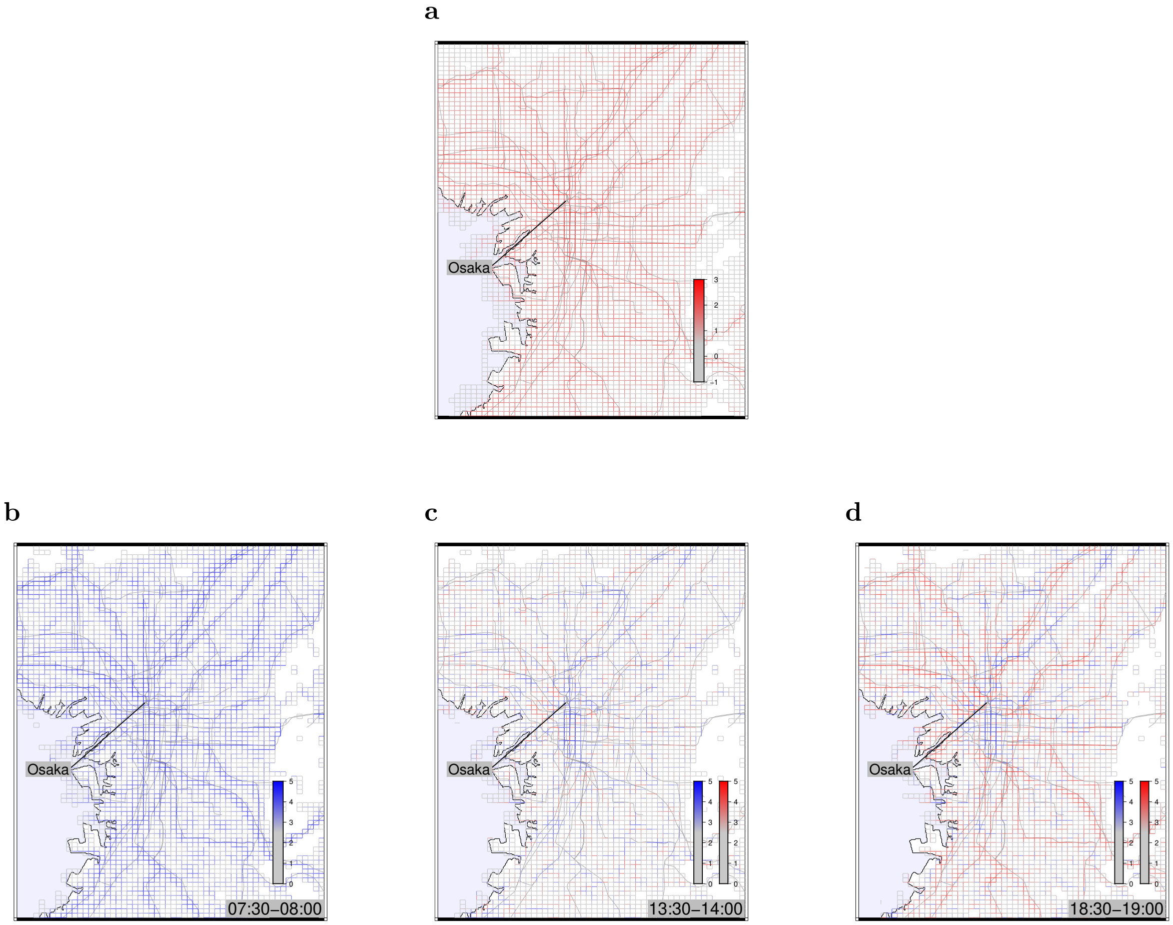

FIG. S4: (a) Volume map of conductance in the Osaka metropolitan area which is drawn on a logarithmic scale. (b-d) Current patterns on the map in the morning, afternoon, and evening. The red lines show that the direction of those currents is different from that in the morning current pattern. S4b. The strength of the flow was plotted on a logarithmic scale. The gray lines in the map represent railways.

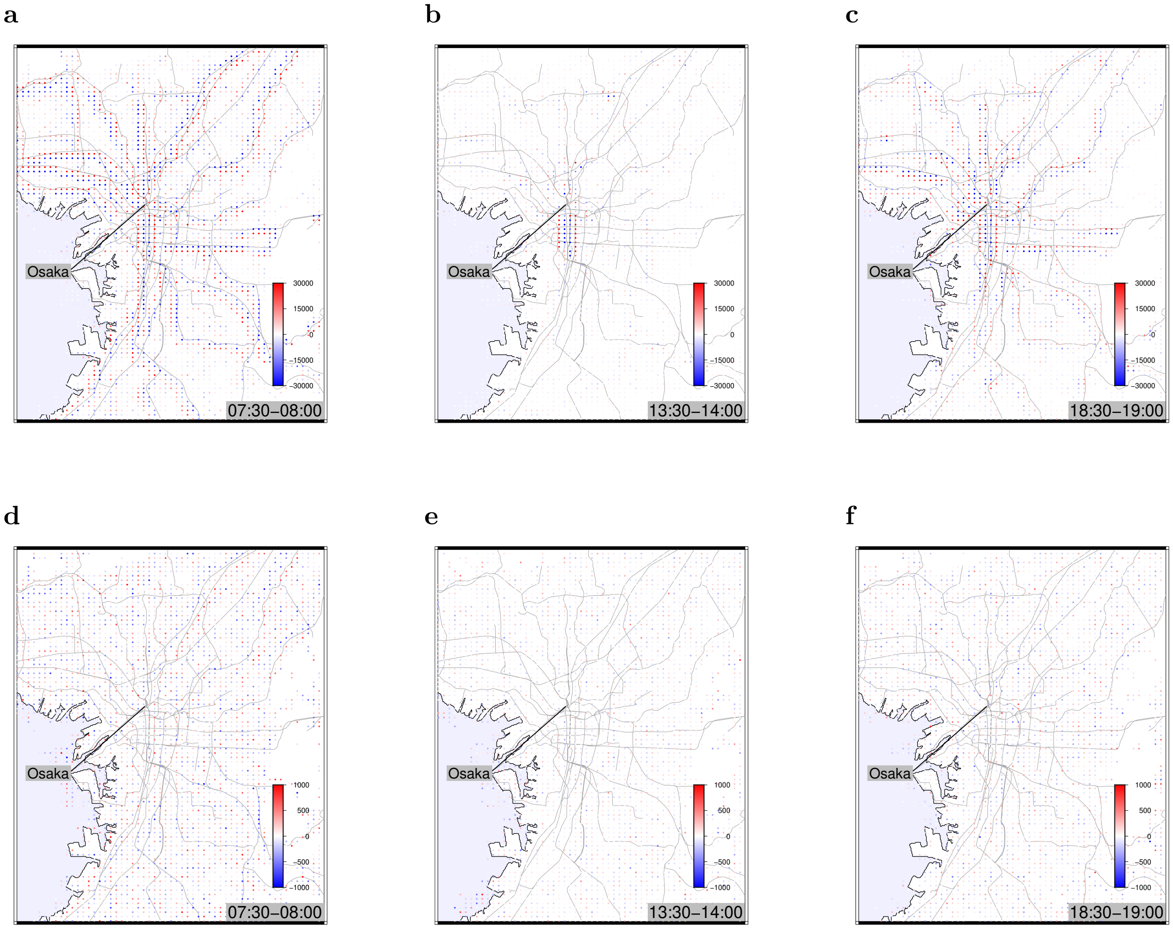

FIG. S5: (a-c) Spatial configuration maps of rotation around the center of Osaka in the morning (7:30-8:00), afternoon (13:30-14:00), and evening (18:30-19:00) at the initial iteration $\tau = 0$. (d-f) Spatial configuration maps of rotation around the center of Osaka in the morning (7:30-8:00), afternoon (13:30-14:00), and evening (18:30-19:00) after optimization.

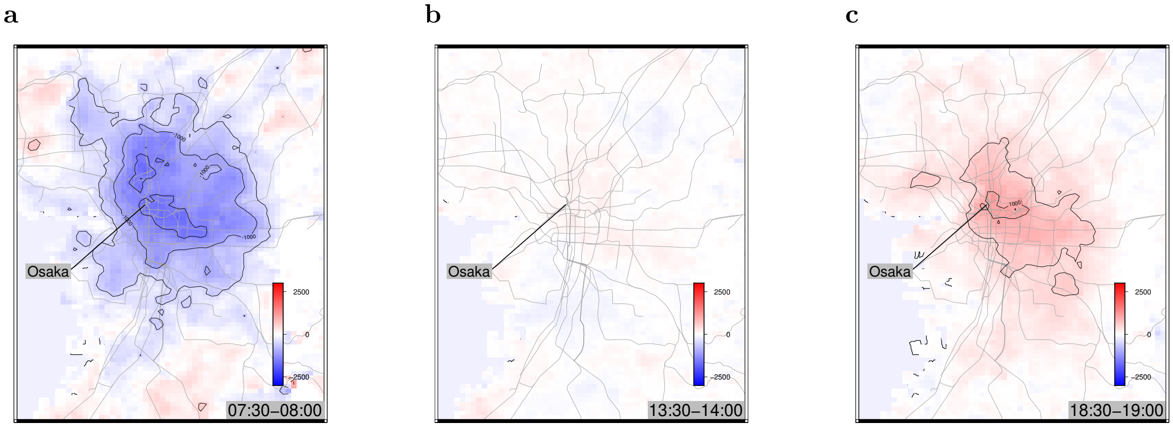

FIG. S6: (a-c) Potential shape around Osaka metropolitan area in the morning, afternoon and evening. Blue and red represent valley-like and mountain-like shapes, respectively.

B. The results around Nagoya metropolitan area

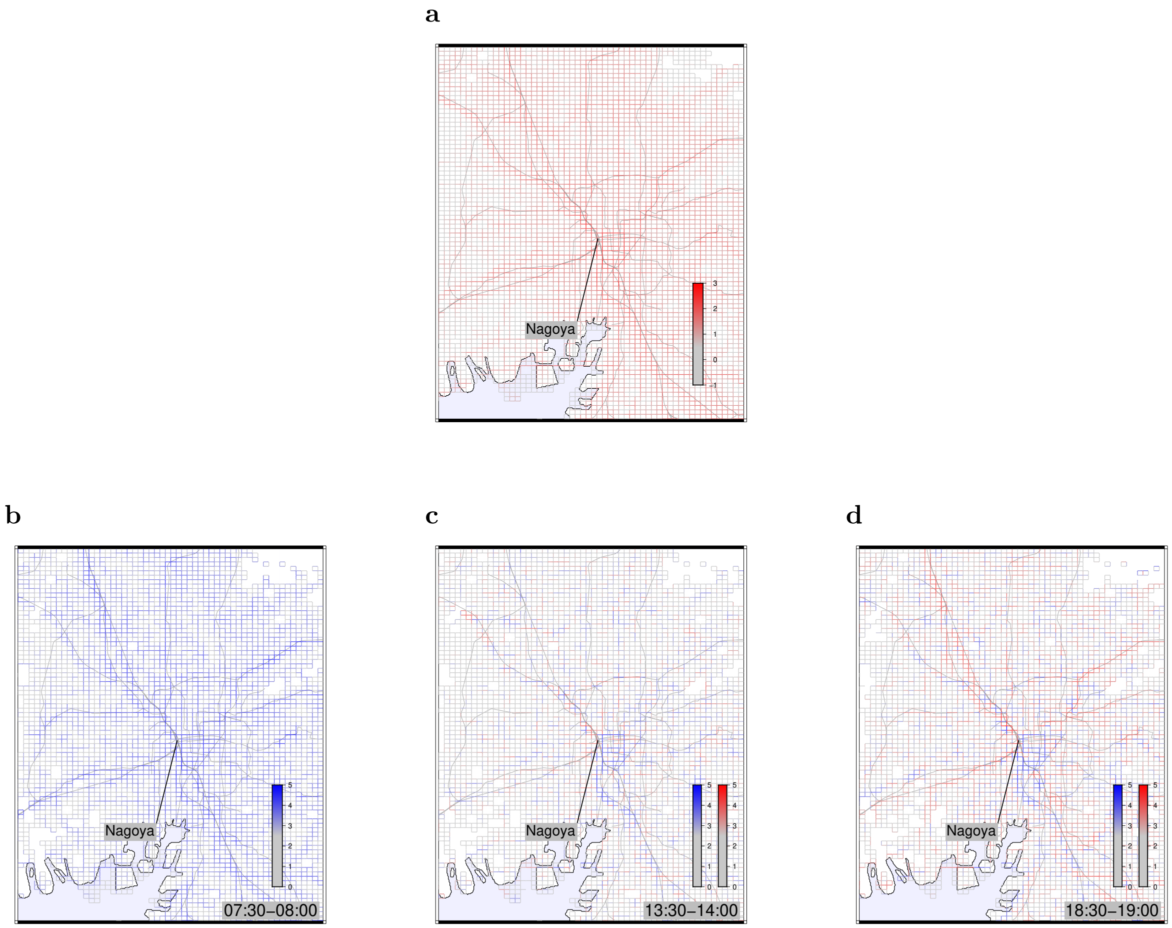

FIG. S7: (a) Volume map of conductance in the Nagoya metropolitan area which is drawn on a logarithmic scale. (b-d) Current patterns on the map in the morning, afternoon, and evening. The red lines show that the direction of those currents is different from that in the morning current pattern. S7b. The strength of the flow was plotted on a logarithmic scale. The gray lines in the map represent railways.

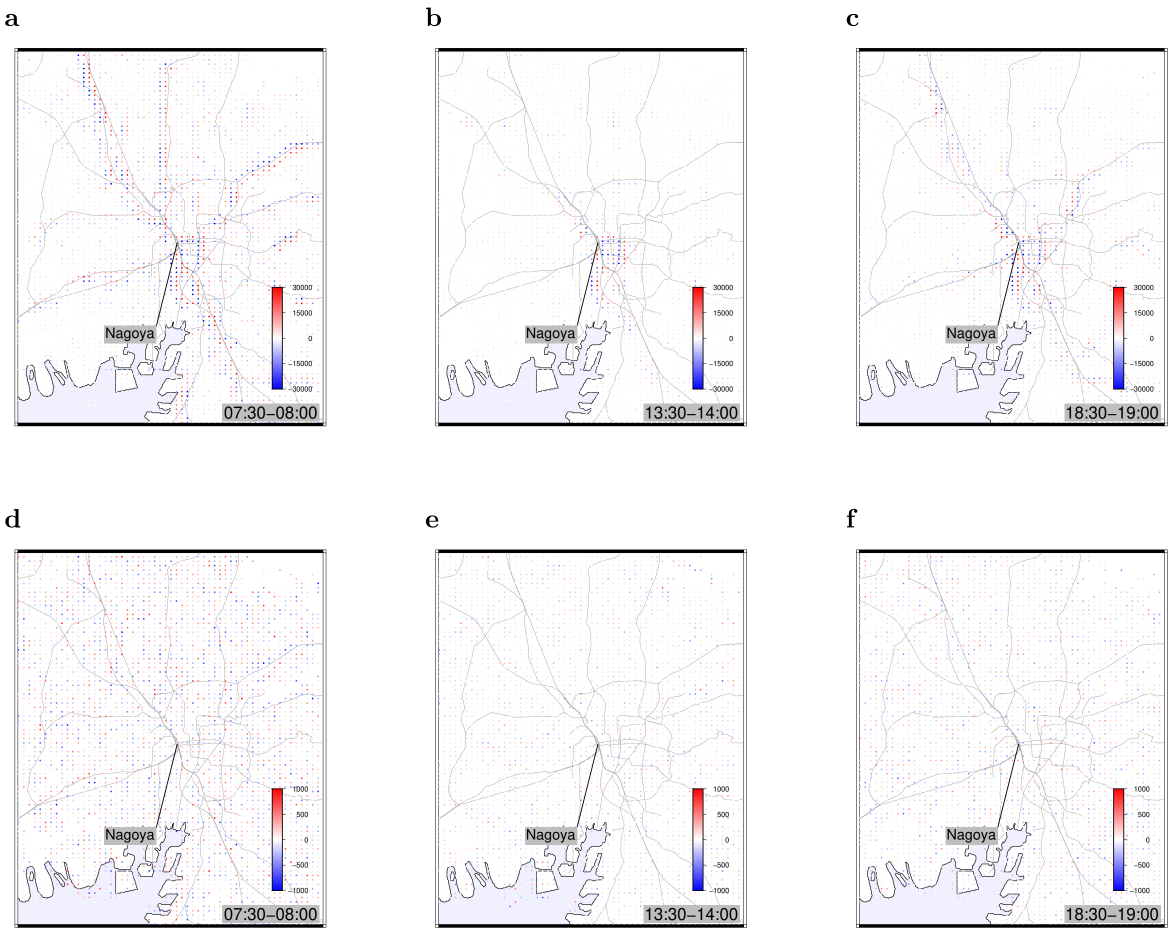

FIG. S8: (a-c) Spatial configuration maps of rotation around the center of Nagoya in the morning (7:30-8:00), afternoon (13:30-14:00), and evening (18:30-19:00) at the initial iteration $\tau = 0$. (d-f) Spatial configuration maps of rotation around the center of Nagoya in the morning (7:30-8:00), afternoon (13:30-14:00), and evening (18:30-19:00) after optimization.

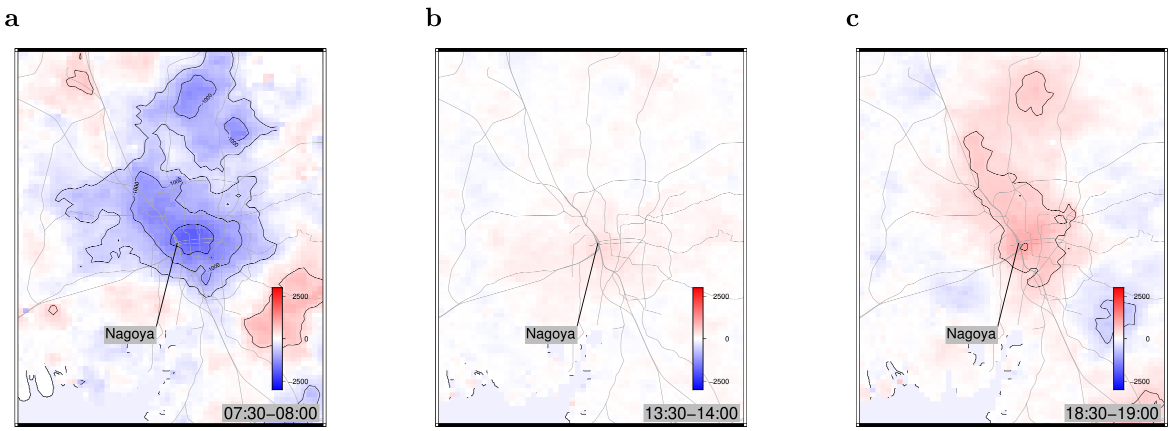

FIG. S9: (a-c) Potential shape around Nagoya metropolitan area in the morning, afternoon and evening. Blue and red represent valley-like and mountain-like shapes, respectively.

VI. FLUCTUATION-DISSIPATION RELATION FOR 3 CITIES

We calculate the daily fluctuations around the mean currents. The mean current from (i,j) to (i',j') at time interval k, denoted $\boldsymbol{I}_{(i,j)\to(i',j'),k}$, is defined by taking the average over the weekdays of the year. The standard deviation of the daily fluctuation is calculated as follows:

$$\sigma_{(i,j)\to(i',j'),k} = \sqrt{\frac{1}{N_{T,(i,j)\to(i',j'),k}} \sum_{T'} (\boldsymbol{I}_{(i,j)\to(i',j'),k} - \boldsymbol{I}_{T',(i,j)\to(i',j'),k})^2}, \tag{5}$$

where $\boldsymbol{I}_{T',(i,j)\to(i',j'),k}$ denotes the current from (i,j) to (i',j') of the time interval k on day T, and $N_{T,(i,j)\to(i',j'),k}$ is the number of days of the current from (i,j) to (i',j') at time interval k. To reduce large fluctuations caused by the small sample size, we excluded 80% of cells with very small user numbers in less populated areas. Fig. S10 is a log-log plot of the variance $\sigma^2_{(i,j)\to(i',j'),k}$ of current fluctuations conditioned by the value of conductance G for all resistors for all time intervals. We find a linear relation between the variance of currents and the conductance, which are nearly the same for 3 big cities. Note that this relation is consistent with the following well-known fluctuation-dissipation theorem of thermal noise for electric circuits, which holds in the case of no mean currents[6]

$$\left\langle V^2 \right\rangle \propto \frac{T_k}{G}, \tag{6}$$

where T_k denotes the temperature in Kelvin. As the voltage V is equal to I/G, this relation is equivalent to the following relation:

$$\left\langle I^2 \right\rangle \propto G. \tag{7}$$

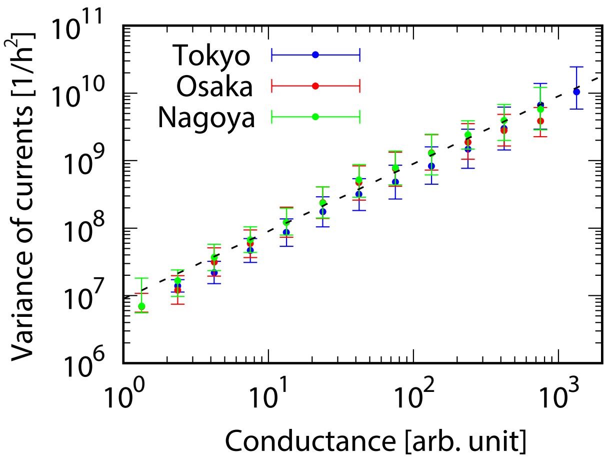

FIG. S10: Relationship between the variance of current in each cell and conductance for 3 cities in Japan with scaling exponent 1.0.

[1] Y. Nesterov, Smooth convex optimization, in *Lectures on convex optimization* (Springer, 2018) pp. 59–137.

[2] D. P. Kingma and J. Ba, Adam: A method for stochastic optimization, arXiv preprint arXiv:1412.6980 (2014).

[3] M. Newman, *Networks* (Oxford university press, 2018).

[4] Geography of tokyo - tokyo metropolitan government, `https://www.metro.tokyo.lg.jp/ENGLISH/ABOUT/HISTORY/history02.htm`, (Accessed on 12/06/2021).

[5] National land numerical information download service, `http://nlftp.mlit.go.jp/ksj-e/index.html`, (Accessed on 11/30/2021).

[6] H. Nyquist, Thermal agitation of electric charge in conductors, Physical review **32**, 110 (1928).
